# In Utero Heat Stress Alters the Offspring Epigenome

**DOI:** 10.1038/s41598-018-32975-1

**Published:** 2018-10-02

**Authors:** A. L. Skibiel, F. Peñagaricano, R. Amorín, B. M. Ahmed, G. E. Dahl, J. Laporta

**Affiliations:** 1 Department of Animal Sciences, University of Florida, Gainesville, FL USA; 2 University of Florida Genetics Institute, University of Florida, Gainesville, FL USA

## Abstract

Exposure to intrauterine heat stress during late gestation affects offspring performance into adulthood. However, underlying mechanistic links between thermal insult in fetal life and postnatal outcomes are not completely understood. We examined morphology, DNA methylation, and gene expression of liver and mammary gland for bull calves and heifers that were gestated under maternal conditions of heat stress or cooling (i.e. in utero heat stressed *vs*. in utero cooled calves). Mammary tissue was harvested from dairy heifers during their first lactation and liver from bull calves at birth. The liver of in utero heat stressed bull calves contained more cells and the mammary glands of in utero heat stressed heifers were comprised of smaller alveoli. We identified more than 1,500 CpG sites differently methylated between maternal treatment groups. These CpGs were associated with approximately 400 genes, which play a role in processes, such as *development*, *innate immune defense, cell signaling*, and *transcription and translation*. We also identified over 100 differentially expressed genes in the mammary gland with similar functions. Interestingly, fifty differentially methylated genes were shared by both bull calf liver and heifer mammary gland. Intrauterine heat stress alters the methylation profile of liver and mammary DNA and programs their morphology in postnatal life, which may contribute to the poorer performance of in utero heat stressed calves.

## Introduction

Early life experiences can program physiological function and health and disease outcomes later in life^1,2^. Perturbations in the intrauterine environment, from maternal malnutrition and stress to elevated body temperature during pregnancy, can induce structural and functional changes in the fetus that may persist through adulthood^3–5^. In several species, maternal heat stress (*i.e*. high ambient temperature) during gestation affects the growth and physiology of the developing young^6–8^. In dairy cattle, calves born to cows exposed to high ambient temperature during late gestation are lighter and shorter at birth and through puberty, have higher morbidity associated with depressed immune competence, and exhibit alterations in energy metabolism and reproductive physiology compared to those born to cows that were actively cooled^6,9–13^. Moreover, the in utero heat stressed female calves (*i.e*. heifers) produce significantly less milk during their first lactation, approximately two years after the developmental insult^10^.

Late gestation is characterized by enhanced muscle growth and fat deposition that contribute to rapid fetal weight gain^14^. In fact, 60% of calf birth weight is attained during the last 2 months of gestation^15^ and, during this time, organs undergo functional maturation in preparation for sustaining life outside the womb^16^. The liver and mammary gland are key organs mediating in utero heat stress effects on physiology and performance in dairy cattle^17,18^. Development of the mammary gland begins in the conceptus and although there is little formation of mammary parenchymal tissue in utero, the rudimentary ductal system upon which the functional alveoli will later form, develops prior to birth^19,20^. The liver is a key metabolic organ, orchestrating carbohydrate, lipid, and amino acid homeostasis to support the energetic demands of growth, maintenance, and production. Moreover, the liver is recognized as an important immune organ^21–23^.

The mechanisms through which in utero heat stress in late gestation affects postnatal liver and mammary function have yet to be elucidated, but could be attributed, in part, to impairment of tissue growth during the organ maturation stage of fetal development. Insults during late gestation can depress cell proliferation in tissues thereby reducing cell number and stunting tissue growth^24,25^. Ahmed *et al*.^26^ reported lower liver weight among in utero heat stressed bull calves at birth. Furthermore, acute exposure to high temperatures in mice induced hepatocyte apoptosis and reduced cell proliferation^27^. In rats, the density of hepatocytes increased concurrent with the number of consecutive days of heat stress exposure, despite general hepatic degeneration^28^. In terms of mammary development, exposure of multiparous dry cows to heat stress depressed mammary epithelial cell proliferation, potentially reducing the total number of cells in the mammary gland capable of producing milk^18^.

Influences of the early life environment on phenotypic variation can be mediated by epigenetic modifications that regulate gene expression. The most stable and well-studied epigenetic mark is DNA methylation. In mammals, methylation occurs predominately at CpG dinucleotides^29^ that can be methylated or demethylated by developmental signals or environmental cues^30,31^. Aberrant methylation patterns occurring during early development may be maintained throughout the life of the animal and permanently alter tissue-specific gene expression and function^32^. The liver and mammary gland are regulated by epigenetic mechanisms that may be altered by heat stress. Milk synthesis and mammary development are regulated by epigenetic processes in the dairy cow^33,34^; global DNA methylation within mammary epithelial cells appears to play a role in mammary cell differentiation and maintenance^33^. In the lactating cow, remethylation of the typically hypomethylated casein gene promoter drastically reduces casein mRNA expression and milk protein synthesis during acute udder infection^35^. DNA methylation also plays an integral role in hepatic differentiation and hepatocyte proliferation^36^. Subjecting male guinea pigs to chronic heat stress alters DNA methylation patterns in the liver of both the F_0_ and F_1_ generations^37^. However, the mechanistic links between developmental insult and mammary and liver function have yet to be fully elucidated. Our hypothesis is that in utero heat stress drives changes in phenotype by altered DNA methylation of key regulatory gene pathways in the liver and mammary gland. Specific objectives of this study were to, 1) examine effects of fetal exposure to intrauterine heat stress during late gestation on the postnatal mammary gland and liver tissue microstructure and, 2) to characterize changes in DNA methylation and gene expression that may explain the observed phenotypic outcomes.

## Results

### Liver and mammary gland histology

**Figure 1:**
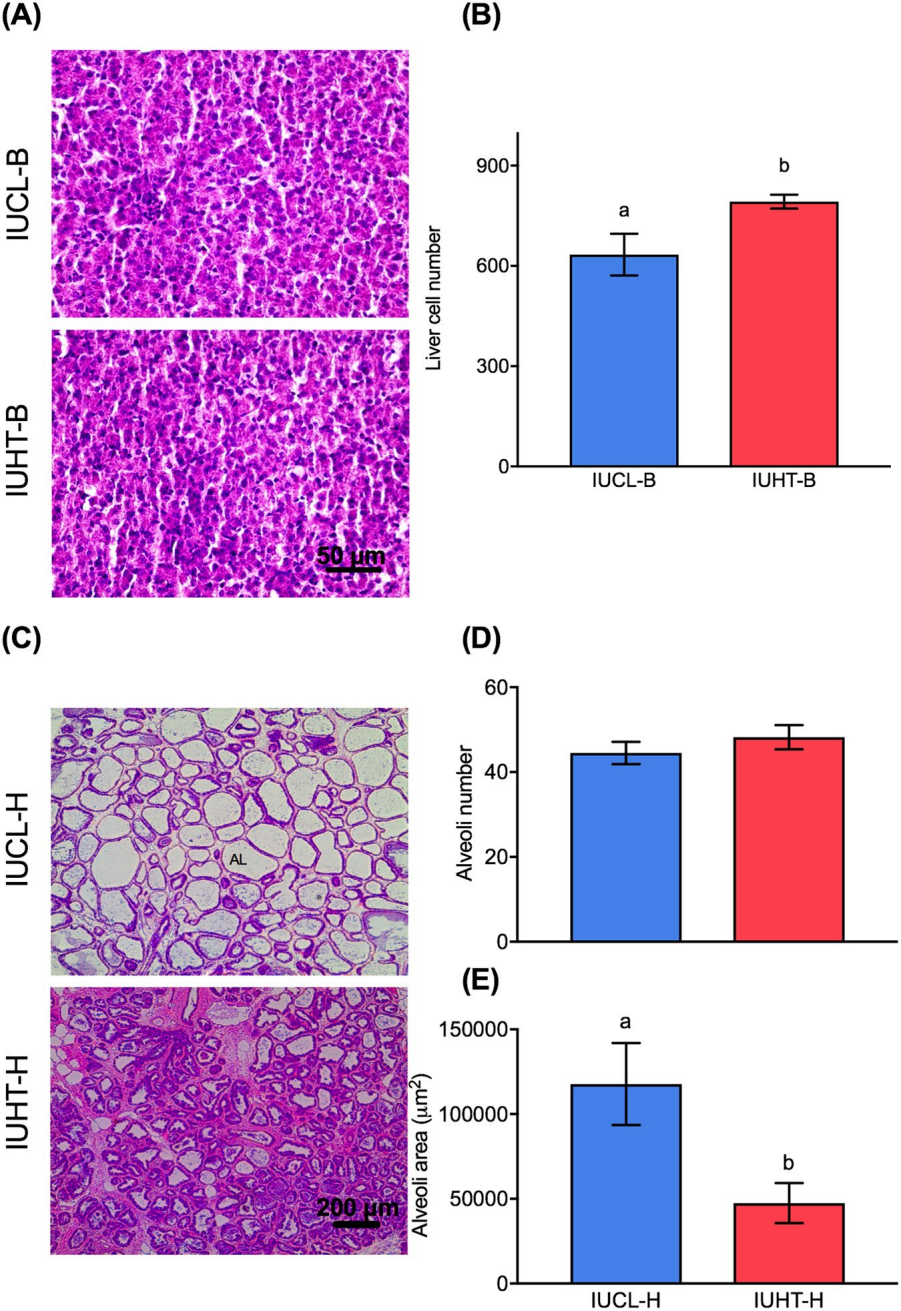
Histological evaluation of liver and mammary gland tissue. Liver was collected from in utero heat stressed (IUHT-B) and in utero cooled (IUCL-B) bull calves at birth. Mammary tissue was collected from in utero heat stressed (IUHT-H) and in utero cooled (IUCL-H) heifers at 21 days into their first lactation. (**A**) Photomicrograph of hematoxylin and eosion (H&E) stained liver tissue at 40x. (**B**) Difference in the number of liver cells between IUHT-B and IUCL-B. The liver of IUHT-B contained more cells than the liver of IUCL-B (*P* = 0.04). (**C**) Photomicrograph of H&E stained mammary tissue at 20x. AL = alveolar lumen. (**D**) Difference in the number of alveoli in the mammary glands of IUHT-H and IUCL-H. Alveoli number was similar between treatment groups (*P* = 0.38). (**E**) Difference in area of alveoli in the mammary glands of IUHT-H and IUCL-H. Alveoli area was larger for IUCL-H relative to IUHT-H (*P* = 0.05). Data are presented as mean ± SEM. Different letters indicate a statistically significant difference.

The liver of in utero heat stressed bull calves (IUHT-B) contained significantly more cells than the liver of in utero cooled bull calves (IUCL-B; 792.2 ± 20.5 vs. 634 ± 62.4, respectively; *t* = 2.41, *df* = 8, *P* = 0.04; Fig. 1A,B). The mammary glands of in utero heat stressed heifers (IUHT-H) and in utero cooled heifers (IUCL-H) had a similar number of alveoli (*t* = 0.95, *df* = 5, *P* = 0.38), but the area of IUCL-H alveoli was significantly larger than IUHT-H (*t* = 2.61, *df* = 5, *P* = 0.05; Fig. 1C–E).

### Differentially methylated genes and gene set enrichment analysis

#### Bull calf liver

**Figure 2:**
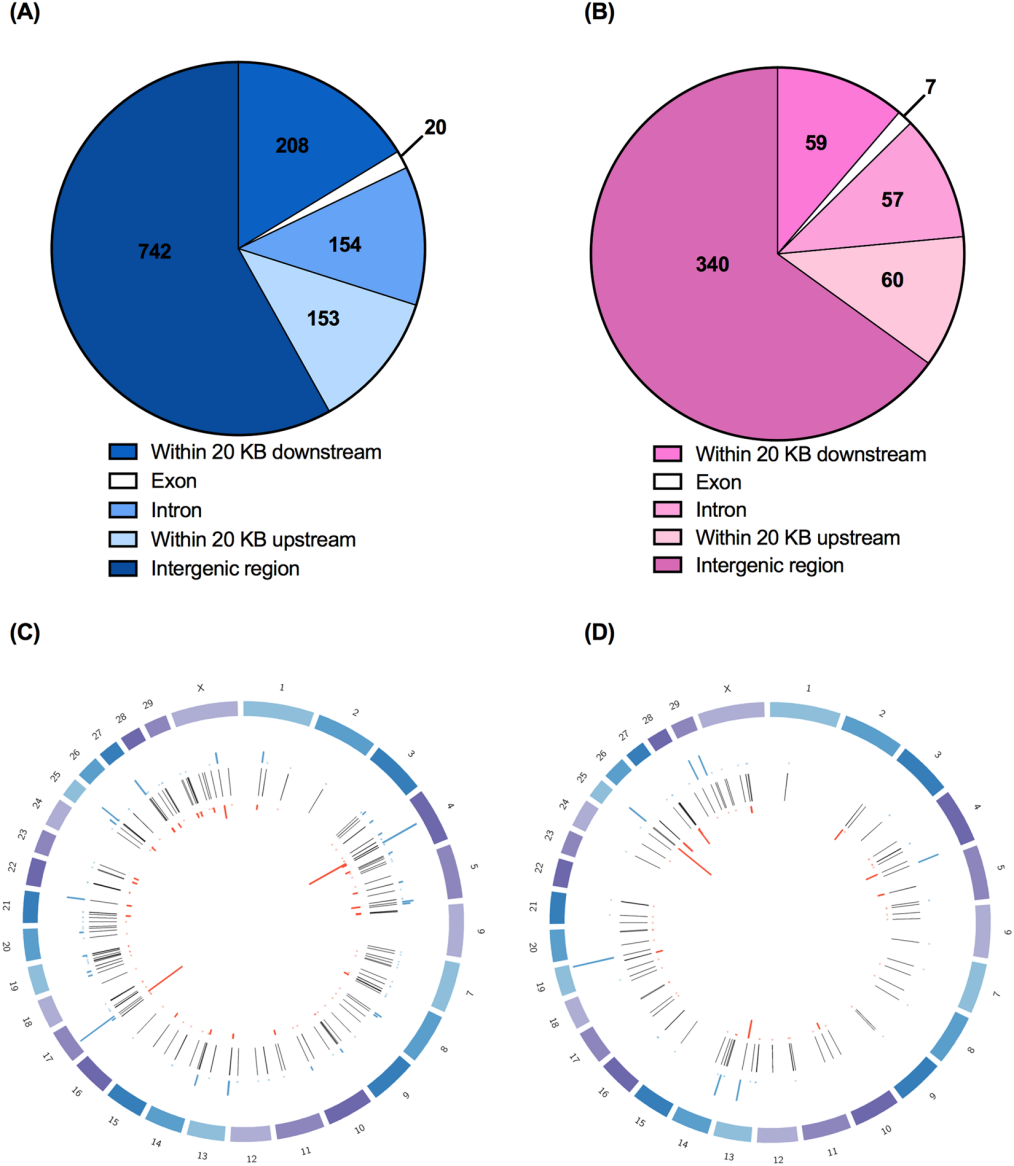
Genomic locations of differentially methylated cytosines (DMCs) and genes (DMGs). Liver tissue was collected from in utero heat stressed (IUHT-B) and in utero cooled (IUCL-B) bull calves at birth. Mammary tissue was collected from in utero heat stressed (IUHT-H) and in utero cooled (IUCL-H) heifers at 21 days into their first lactation. (**A**) Genomic locations of DMCs for bull calf liver; (**B**) Genomic locations of DMCs for heifer mammary gland. Cut-off criteria for defining DMCs was 15% methylation difference between treatment groups and *q*-value < 0.2. DMCs were located within 20 KB upstream of the transcription start site of a gene, within introns, within exons, or within 20 KB downstream of the gene end. DMCs more than 20 KB upstream or more than 20 KB downstream of a gene were considered to reside in the intergenic region. (**C**) Chromosomal location of DMGs and DMCs for bull calf liver; (**D**) Chromosomal location of DMGs and DMCs for heifer mammary gland. Outer circle represents the chromosomes where DMCs and DMGs are located. The middle circle of black bars represents DMGs. DMGs contain at least 1 DMC. The red and blue bars indicate cytosines that are hypo-and hypermethylated in the heat stressed group relative to the cooled group, respectively. Height of the bars corresponds to the number of DMCs.

In liver tissue of IUCL-B, 421 million cytosines were identified in the CpG context and 78.1% of those cytosines were methylated. For IUHT-B, there were 243 million cytosines in the CpG context, 79.5% of which were methylated. Based on our cut-off criteria of ≥15% methylation difference and *q*-value ≤ 20%, there were 1,277 differentially methylated cytosines (DMCs) between IUCL-B and IUHT-B. Principal component analysis based on DMCs showed clear separation into in utero heat stressed and in utero cooled groups (Supplementary Fig. S1). Location of DMCs were identified as within a gene (intron or exon), within 20 Kb upstream of the transcription start site of a gene, or within 20 Kb downstream of the gene end. DMCs beyond 20 KB upstream or downstream of the gene were considered to be in the intergenic region. The majority of DMCs resided in the intergenic regions (n = 744), but a substantial number were associated with other genomic features (Fig. 2A). Of the 153 DMCs within 20 Kb upstream of the transcription start site, 68 are within 10 Kb upstream and 35 were within 5 Kb upstream. Of the 208 DMCs downstream of the gene end, 152 were within 10 Kb downstream and 91 were within 5 Kb downstream. The 1,277 DMCs comprised 239 differentially methylated genes (DMGs) that were distributed across most of the bovine chromosomes (Fig. 2C, Supplementary Table S1). DMGs included 16 non-coding RNAs, 38 encoding rRNAs (30 code for 5S rRNA and 8 code for 5.8S rRNA), and 43 novel bovine genes. Seven of the novel bovine genes are pseudogenes and the rest are protein-coding genes. A few interesting DMGs between IUHT-B and IUCL-B liver are advanced glycosylation end-product specific receptor (*AGER*), mediator complex subunit 1 (*MED1*), and BCL2 associated athanogene 1 (*BAG1*).

**Figure 3:**
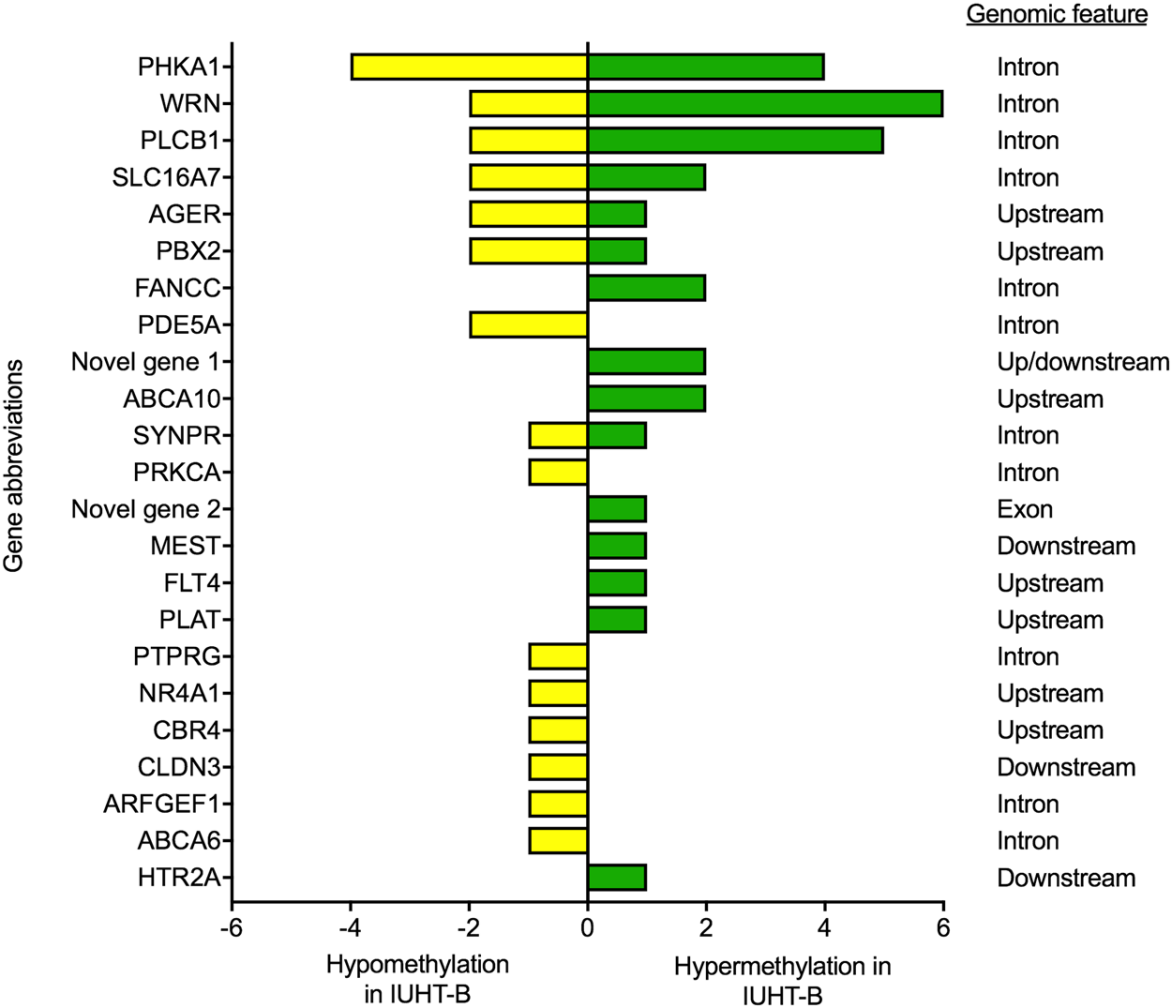
Number and direction of differentially methylated cytosines (DMCs) corresponding to differentially methylated genes (DMGs) in the liver of in utero heat stressed (IUHT-B) compared to in utero cooled (IUCL-B) bull calves. DMGs are of the significant pathways and biological functions (from Table 1), identified through enrichment analysis. Hypomethylated cytosines are indicated by the yellow bars. Green bars denote hypermethylated cytosines. Hypo- or hypermethylation of cytosines refers to the in utero heat stressed group relative to the in utero cooled group.

Based on enrichment analyses, the significant pathways and functions for DMGs included *transcription, immune function, cell signaling, enzyme activity, cell cycle*, and *development* (Table 1). Among DMGs in these pathways, some genes contained cytosines that were hypomethylated in IUHT-B compared to IUCL-B, some only hypermethylated cytosines, and some contained both hypo- and hypermethylated cytosines (Fig. 3, Supplementary Table S2). For example, there were 3 DMCs upstream of the *AGER* gene; 1 DMC was hypermethylated whereas 2 cytosines were hypomethylated in the liver of IUHT-B relative to IUCL-B.

**Table 1:** Significant pathways, biological functions, and molecular processes of differentially methylated genes in the liver of in utero heat stressed (IUHT-B) compared to in utero cooled bull calves (IUCL-B).

Pathways and functions	Genes in pathway	Differentially methylated genes	*P*-value
Transcription factors	188	*PTPRG, NOVEL GENE 1, NOVEL GENE 2, SYNPR, PDE5A*	0.03
Immunologic receptors	48	*AGER, PRKCA, SYNPR, PTPRG*	<0.001
Positive regulation of MAPK cascade	105	*PDE5A, HTR2A, PLCB1, PRKCA*	0.02
Calcium signaling pathway	163	*HTR2A, PHKA1, PLCB1, PRKCA*	*0.07*
Gap junction	83	*PRKCA, PLCB1, HTR2A*	0.04
Regulation of nucleotide metabolic process	62	*PRKCA, HTR2A, PDE5A*	0.02
Fat cell differentiation	57	*NR4A1, PLCB1, HTR2A*	0.02
Cell cycle proteins	67	*FANCC, WRN, NOVEL GENE 2*	0.02
ABC transporters	41	*ABCA6, ABCA10*	*0.06*
Positive regulation of glycoprotein biosynthetic pathway	6	*PLCB1, ARFGEF1*	0.001
Protein heterooligomerization	13	*CBR4, CLDN3*	0.007
Mesoderm development	20	*MEST, NOVEL GENE 2*	0.02
Phosphoric diester hydrolase activity	32	*PLCB1, PDE5A*	0.04
Major histocompatibility complex	20	*AGER, PBX2*	0.01
Zinc fingers	33	*SYNPR, PTPRG*	0.03
RNA splicing	37	*PRKCA, NOVEL GENE 2*	0.04
Sodium-hydrogen antiporter	8	*PLCB1, SLC16A7*	0.002
Phosphatidylinositols	12	*PLCB1, HTR2A*	0.005
Urokinase-type plasminogen activator	15	*PLAT, FLT4*	0.008
Type C phospholipase	23	*PLCB1, AGER*	0.02
Interleukin-8	29	*SYNPR, PTPRG*	0.03

Note: Enrichment analysis of differentially methylated genes was achieved through the Kyoto Encyclopedia for Genes and Genomes (KEGG) database, Medical Subject Headings (MeSH) database, and Gene Ontology Consortium database. *P*-values are derived from Fisher’s exact tests and indicate significance of enrichment. Italics indicate tendency for enrichment.

#### Heifer mammary gland

**Figure 4:**
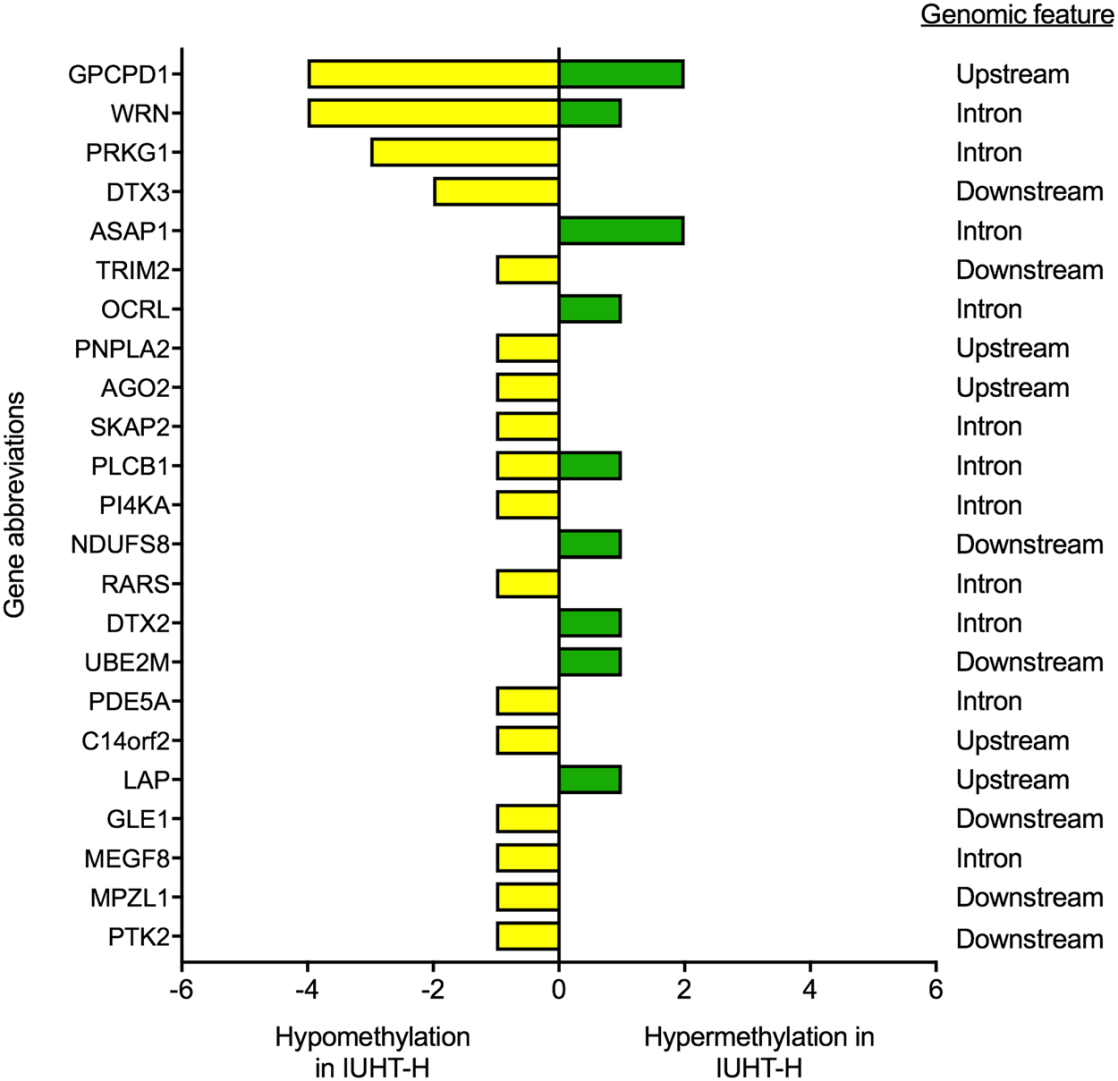
Number and direction of differentially methylated cytosines (DMCs) corresponding to differentially methylated genes (DMGs) in the mammary gland of in utero heat stressed (IUHT-H) compared to in utero cooled (IUCL-H) heifers. DMGs are of the significant pathways and biological functions (from Table 2), identified through enrichment analysis. Hypomethylated cytosines are indicated by the yellow bars. Green bars denote hypermethylated cytosines. Hypo- or hypermethylation of cytosines refers to the in utero heat stressed group relative to the in utero cooled group.

We observed 245 million and 237 million cytosines in CpG enriched areas of mammary DNA for IUCL-H and IUHT-H groups, respectively. The percent of cytosine methylation at CpGs was similar between IUCL-H and IUHT-H (IUCL-H, 74.1%; IUHT-H, 74.8%). There were 523 DMCs between treatment groups (methylation difference ≥15%, *q*-value ≤ 20%). Principle components analysis showed a slight separation between IUCL-H and IUHT-H groups (Supplementary Fig. S1). DMCs were found within the gene body, and upstream or downstream of the gene, but mostly occurred in the intergenic regions (n = 340; Fig. 2B). Of the 60 DMCs within 20 Kb upstream of the transcription start site, 34 were within 10 Kb upstream and 17 were within 5 Kb upstream. Of the 59 DMCs downstream of the gene end, 40 were within 10 Kb downstream and 18 were within 5 Kb downstream. The 523 DMCs corresponded to 135 DMGs spread across the genome (Fig. 2D, Supplementary Table S1), including 7 non-coding RNAs, 19 genes for 5S rRNA, 8 genes for 5.8S rRNA, and 23 novel bovine genes. Three of the novel genes are non-coding RNAs, 4 are pseudogenes, and 16 are protein coding. A few interesting DMGs between IUHT-H and IUCL-H mammary gland were cGMP dependent protein kinase 1 (*PRKG1*), phospholipase C beta 1 (*PLCB1*), and tripartite motif genes (*TRIM 28* and *TRIM 37*). Significant pathways and biological functions identified through enrichment analysis included *protein binding, phosphorylation, enzyme and cell activation*, and *cell signaling* (Table 2). DMGs within these pathways varied in the number of DMCs they contained and the direction of methylation difference (Fig. 4, Supplementary Table S2).

**Table 2:** Significant pathways, biological functions, and molecular processes of differentially methylated genes in the mammary gland of in utero heat stressed (IUHT-H) versus in utero cooled heifers (IUCL-H).

Pathways and functions	Genes in pathway	Differentially methylated genes	*P*-value
Protein binding	625	*ASAP1, C14orf2, PDE5A, PI4KA, PRKG1, WRN, MEGF8, NOVEL GENE 3*	0.006
Phosphorylation	351	*PDE5A, PRKG1, ASAP1, MPZL1, PTK2*	0.02
Enzyme activation	273	*PRKG1, PTK2, ASAP1, PLCB1*	0.03
Cell activation	177	*PLCB1, PRKG1, SKAP2, PDE5A*	0.02
Hydrolase activity, acting on ester bonds	243	*AGO2, PNPLA2, PLCB1, PDE5A*	0.04
Inositol phosphate metabolism	54	*PLCB1, PIK4A, OCRL*	0.003
Tyrosine	88	*MPZL1, PTK2, NDUFS8*	0.007
Adenosine triphosphate	120	*C14orf2, PRKG1, WRN*	0.01
Ligase activity	111	*TRIM2, UBE2M, RARS*	0.02
Regulation of GTPase activity	112	*ASAP1, PLCB1, PRKG1*	0.02
Notch signaling pathway	39	*DTX2, DTX3*	0.02
Gap junction	83	*PLCB1, PRKG1*	*0.08*
Negative regulation of cell-cell adhesion	19	*PDE5A, PRKG1*	0.005
Regulation of translational initiation	31	*AGO2, GLE1*	0.01
cGMP binding	10	*PDE5A, PRKG1*	0.001
Lipase activity	29	*PLCB1, PNPLA2*	0.01
Allosteric site	16	*PDE5A, PRKG1*	0.002
Src homology domains	17	*MPZL1, ASAP1*	0.002
Cation transport proteins	21	*PLCB1, MEGF8*	0.004
Phosphotyrosine	25	*PTK2, ASAP1*	0.005
Phosphoric diester hydrolases	26	*GPCPD1, PDE5A*	0.006
Src-family kinases	34	*PTK2, ASAP1*	0.01
Cyclic GMP	39	*PDE5A, PRKG1*	0.01
Calcium channels	43	*PLCB1, PRKG1*	0.02

Note: Enrichment analysis of differentially methylated genes was achieved through the Kyoto Encyclopedia for Genes and Genomes (KEGG) database, Medical Subject Headings (MeSH) database, and Gene Ontology Consortium database. *P*-values are derived from Fisher’s exact tests and indicate significance of enrichment. Italics indicate tendency for enrichment.

### Shared DMGs between bull calf liver and heifer mammary tissue

**Figure 5:**
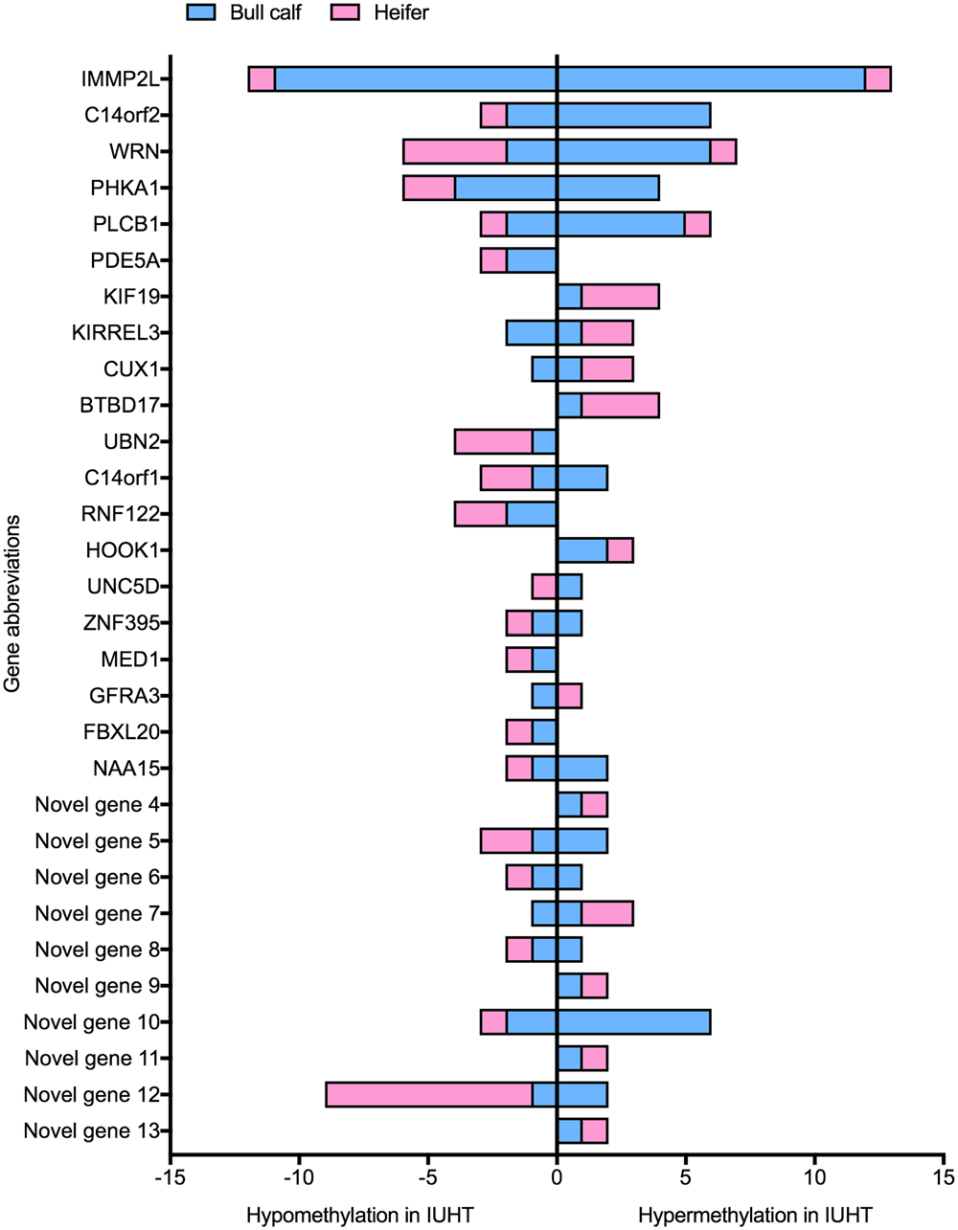
Number and direction of differentially methylated cytosines (DMCs) corresponding to 30 shared differentially methylated genes (DMGs) between liver and mammary gland tissue. Liver was collected from in utero heat stressed (IUHT-B) and in utero cooled (IUCL-B) bull calves at birth. Mammary tissue was collected from in utero heat stressed (IUHT-H) and in utero cooled (IUCL-H) heifers at 21 days into their first lactation. Methylation comparison was made between IUHT and IUCL groups for bull calves and heifers separately. Hypo- or hypermethylation of cytosines refers to IUHT relative to IUCL. Pink bars denote heifer mammary gland, blue bars indicate bull calf liver. Novel genes are genes that have not yet been characterized in the bovine genome. In addition to the 30 shared DMGs shown here, there were an additional 20 shared DMGs that code for rRNAs.

There were 50 common genes that were differentially methylated both between IUHT-B and IUCL-B and between IUHT-H and IUHT-CL (Fig. 5). Functions of the DMGs included mitochondrial function and oxidative defense (*IMMP2L*, *C14orf2*), DNA repair (*WRN*), cell signaling (*PLCB1*, *PDE5A*), transcription (*CUX1*, *ZNF395*, *MED1*), intracellular transport (*KIF19*, *HOOK1*), and cell adhesion and migration (*UNC5D*, *KIRREL3*). Additionally, 12 of the shared DMGs code for 5S rRNA, 8 code for 5.8S rRNA, and 10 are novel bovine genes (Supplementary Table S3). Of the novel bovine genes, 8 are protein-coding genes and 2 are pseudogenes.

### Differentially expressed genes and gene set enrichment analysis

We identified 15,366 genes expressed in the mammary gland of heifers, 117 of which were differentially expressed between IUHT-H and IUCL-H (*P* ≤ 0.01; Supplementary Table S4). Major functions of the differentially methylated genes (DEGs) included immunity, metabolism, cell signaling, and development (Supplementary Table S5). One of the DEGs is a non-coding RNA and 16 are novel bovine genes. Of the novel bovine genes, 15 are protein-coding genes and 1 is a pseudogene.

### Correlation between methylation and gene expression

**Figure 6:**
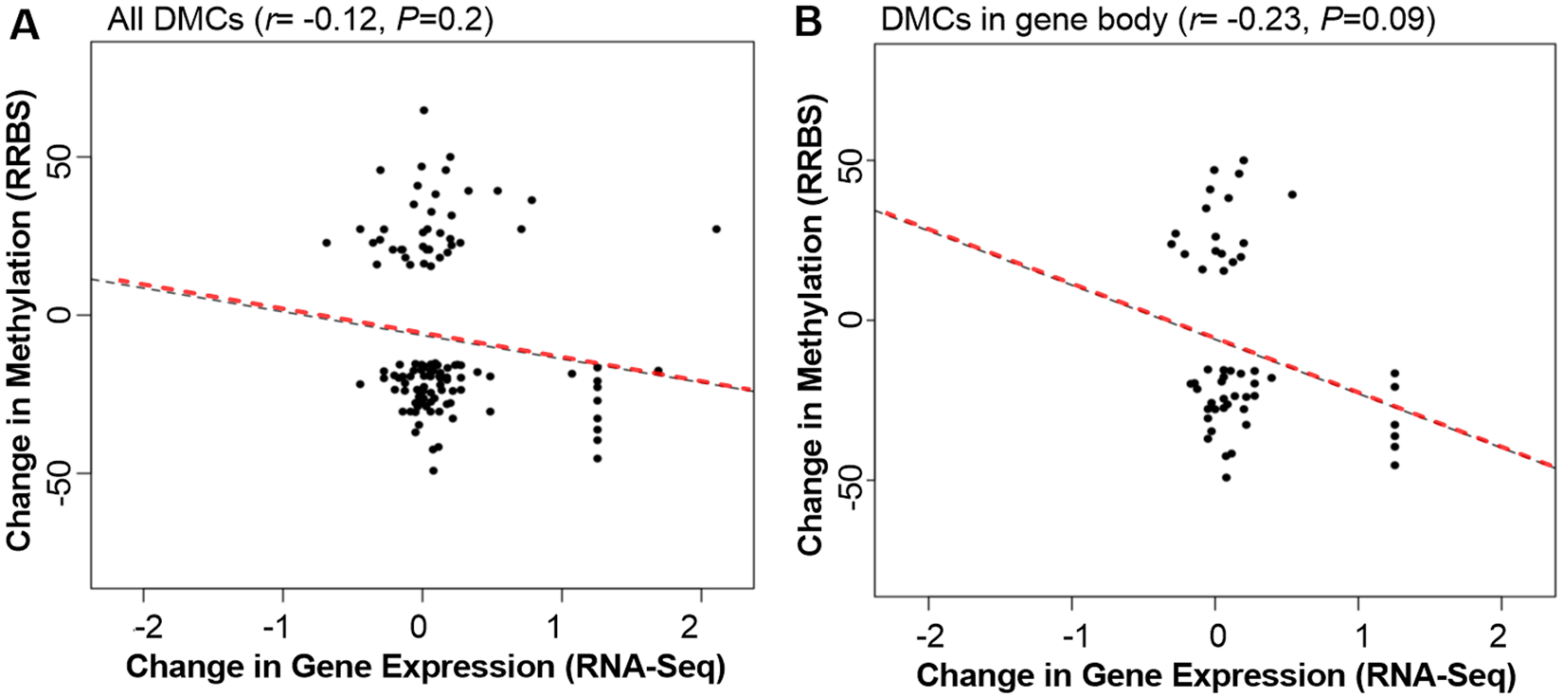
Relationship between differential cytosine methylation and differential gene expression in the mammary gland of in utero heat stressed (IUHT-H) and in utero cooled (IUCL-H) heifers. (**A**) Overall DMCs regardless of their genomic location. There was a non-significant negative correlation between differential methylation and differential gene expression when including DMCs regardless of genomic location (*r* = −0.12, *P* = 0.2). (**B**) Includes only DMCs located in the gene body. There was a tendency for a significant negative correlation between differential methylation and differential gene expression when including only DMCs located in the gene body (*r* = −0.23, *P* = 0.09).

There was a non-significant negative correlation between change in DNA methylation and change in gene expression between IUHT-H and IUCL-H (*r* = −0.12, *P* = 0.2; Fig. 6A) and a tendency for a significant negative correlation between change in DNA methylation and change in gene expression when only including DMCs located in the gene body (*r* = −0.23, *P* = 0.09; Fig. 6B). There was no significant relationship between differential methylation of DMCs upstream or downstream of the gene and gene expression.

## Discussion

In dairy cattle, exposure of the pregnant cow to environmental heat stress impacts her calf up to at least two years after the insult. In particular, calves born to heat stressed dams have reduced immune function, altered systemic metabolism, and reduced milk production during the first lactation^6,9–13^. The underlying mechanisms responsible for phenotypic effects of in utero heat stress, however, are still unknown. Herein we examined DNA methylation and gene expression changes in the mammary gland as potential mechanisms modulating in utero heat stress effects on offspring. We identified approximately 400 genes that were differentially methylated and approximately 100 genes that were differentially expressed between in utero heat stressed and in utero cooled cattle, corroborating previous documentation of heat induced changes in DNA methylation. Two paternal imprinted genes in mouse blastocysts were demethylated upon acute exposure to high temperature^38^. Wild guinea pig males exposed to chronic high temperature showed changes in hundreds of methylated regions in the liver after heat exposure^37^. Furthermore, differences in methylation levels in liver and testis were detected in sons conceived before and after paternal exposure to heat, indicative of transgenerational epigenetic inheritance^37^.

Based on the differentially methylated genes in bull calf liver, enrichment analysis revealed several pathways and biological functions potentially epigenetically altered by in utero exposure to heat stress, such as immune function, gene and protein expression, including posttranscriptional and posttranslational modifications, and cell processes. Immune related pathways, functions, and terms included *immunologic receptors*, *major histocompatibility complex*, and *interleukin-8*. The *AGER* gene appears in multiple immune related pathways and encodes a receptor for advanced glycation end products (RAGE), a pattern recognition receptor belonging to the immunoglobulin superfamily^39–41^. RAGE is involved in a number of intracellular signaling pathways that regulate cell functions, such as proliferation, apoptosis, autophagy, cell migration, and inflammation^42^. Binding of certain ligands to RAGE can also generate reactive oxygen species by activation of NADPH oxidase^43^ and results in the cellular depletion of glutathione and ascorbic acid, two important antioxidants^44^, which may contribute to oxidative stress. A recent proteomics analysis of the liver of periparturient cows heat stressed during late gestation found evidence of oxidative stress^17^. In the present study, genes contributing to superoxide metabolism, such as *NOXO1*, *GSTT1*, and *SOD2*, were upregulated in the mammary gland of IUHT-H relative to IUCL-H. Higher levels of pro-inflammatory cytokines have been detected in peripheral blood mononuclear cells of heat stressed pregnant cows and in utero heat stressed calves have higher circulating acute phase proteins, whose production is induced by pro-inflammatory cytokines^45,46^. Together, these results suggest that altered DNA methylation patterns may contribute to heightened inflammatory responses and oxidative stress among in utero heat stressed cattle.

Several genes involved in chromatin structure and the transcriptional machinery were differentially methylated in the liver of IUHT-B versus IUCL-B, including *MED1* encoding a subunit of a transcription activator^47^, *H2AY* encoding a core histone^48^, and *EPOP*, which regulates chromatin structure at actively transcribed genes^49^. Three genes in the zinc finger gene family, *ZMAT5*, *ZNF608*, and *ZNF395* were differentially methylated in the liver. Many zinc fingers function as transcription factors, binding specific long sequences of DNA to control gene transcription^50–52^. *ZNF395*, specifically, appears to play a role in innate immunity, through a hypoxia-induced inflammatory response and through an antiviral pathway mediated by NF-κB activation. Interestingly, both NF-κB activation and *ZNF395* expression are induced by hypoxia and *ZNF395* is a target gene of HIFα, an integral mediator of the cellular response to hypoxia^53–56^. Heat stress during gestation reduces placental mass and vascularization, leading to lower oxygen and nutrient delivery to the conceptus and intrauterine growth restriction^8,57–59^. Thus, it is conceivable that fetal methylation of *ZNF395* in the liver is altered by heat stress-induced intrauterine hypoxia, which may have implications for innate immune function.

Calves born to dams heat stressed during late gestation had a greater number of cells in the liver relative to those born to cooled dams. Similarly, rats exposed to heat stress over several consecutive days had greater hepatocyte density^28^. Differential methylation of genes involved in apoptosis and proliferation, such as *BAG1* and *PRKCA*^60–63^, may be associated with heat stress-induced effects on tissue and organ growth as organ size is determined by regulation of these cell processes. The weight of several organs, including the liver, was lighter at birth in utero heat stressed bull calves^26^. Given that organ mass is a product of cell number as well as cell mass and the mass of the extracellular compartment^64^, the in utero heat stressed calves likely have smaller cells and/or reduced extracellular matrix. Although hepatocytes account for approximately 80% of liver mass^65,66^, it is unclear if the greater hepatic cell number in heat stressed bull calves in our study is due to the presence of more hepatocytes or other cell populations, such as stellate cells or immune cells.

The majority of pathways and functions of differentially methylated genes in heifer mammary gland were related to protein activity and cell signaling. Terms included *phosphorylation*, *hydrolase activity*, *ligase activity*, *GTPase activity*, *lipase activity*, *enzyme activation*, and *protein binding*. DMGs in these pathways, such as *PRKG1*, *PI4KA*, *PLCB1, ASAP1*, and *PTK2*, regulate a variety of physiological processes involved in mammary morphogenesis and milk synthesis^67–73^. *PRKG1*, *PI4KA*, and *PLCB1* regulate intracellular calcium concentration. Calcium is an essential ion for functions such as milk synthesis, immune activation, cell proliferation, and signal transduction. *PI4KA* and *PLCB1* encode enzymes in phosphoinositol biosynthetic pathways, indirectly affecting intracellular calcium release through formation of inositol 1,4,5-trisphosphate (IP_3_), an integral second messenger^67,68^. PLCB1 also catalyzes the formation of diacylglycerol (DAG), another important second messenger^67^. The phosphoinositide pathway is one pathway mediating the mitogenic activity of PRL in mammary epithelial cells^69^. On the other hand, *PRKG1* encodes cGMP dependent protein kinase 1, an intracellular receptor for cGMP, which negatively regulates the release of intracellular calcium by inhibiting IP_3_-mediated Ca release^70^. Results of RNA sequencing and histological evaluation of the mammary gland lend further support for in utero heat stress effects on these pathways and on mammary development. Cell signaling and protein activity were main functions enriched in our dataset of DEGs and included *hydrolase activity, G-protein couple receptor signaling pathway, signaling receptor binding*, and *phospholipase C activity*. Several genes involved in development and organogenesis (*HOXA9, ELLE3, SFRP1, RIPPLY3*, and *FR2B*) and in the phosphoinositide pathway (*SDCBP2* and *INPP5E*), were upregulated in the mammary gland of IUHT-H compared to IUCL-H. We also found evidence of blunted mammary development associated with in utero heat stress; IUHT-H had significantly smaller mammary alveoli relative to IUCL-H at 21 days into the first lactation. Our results suggest that changes in DNA methylation of genes involved in mammary development and milk synthesis may be associated with ultrastructural differences in the mammary gland and with differential milk production observed between IUHT-H and IUCL-H^10^.

For mammary tissue, genes involved in transcription, translation, and gene regulation were differentially methylated between IUHT-H compared to IUCL-H. DMGs with these functions included the zinc finger genes *ZNF395*, *ZC3H6*, *MZF1*, and *ZNF75D AGO2*, and tripartite motif (*TRIM*) genes (*TRIM28* and *TRIM37*). The *AGO2* gene plays a role in transcriptional and post-transcriptional gene silencing^74,75^ and *TRIM* genes regulate gene expression through a variety of mechanisms, such as recruiting transcription coregulators, histone deacetylases, and other chromatin modifiers^76,77^. Several zinc finger genes were also differentially expressed between IUHT-H and IUCL-H; one was downregulated (*ZBTB47*) and three were upregulated (*ZNF555, ZNF134, ZNF177*) in IUHT-H relative to IUCL-H.

Although immune function was not a term significantly enriched in our DMG dataset for mammary tissue, many of the identified DEGs are involved in immune related functions (*immune system process, immunoglobulin production, antigen processing and presentation*, *etc*). Most DEGs in these pathways were upregulated (*BLA-DQB, JCHAIN, DEFB, GRO1, HP*) in the mammary glands of IUHT-H compared to IUCL-H, although a couple were downregulated (*BOLA-DQB*, *BOLA*). These results are in concordance with immune deficits and elevated acute phase protein production observed among in utero heat stressed cattle^26,45,78^, but suggest that DNA methylation is not contributing substantially to regulation of immune-related genes in the mammary gland.

Fifty genes were differentially methylated between IUHT-B and IUCL-B liver and also between IUHT-H and IUCL-H mammary gland. Among these were the DNA repair gene, *WRN*, and two genes encoding mitochondrial proteins, *IMMP2L* and *C14orf2*, important for oxidative defense and ATP synthesis, respectively^79,80^. Furthermore, *ZNF395*, *MED1*, and the developmentally important homeobox gene, *CUX1*, were differentially methylated in both liver and mammary tissue. In both the heifer and bull calf, genes in the notch signaling pathway were differentially methylated (heifer: *DTX2, DTX3*; bull calf: *Notch3, Notch4*), which is involved in cell differentiation and development of both liver and mammary gland^81,82^. Twenty differentially methylated genes shared between bull calf liver and heifer mammary gland were rRNA genes (5S rDNA and 5.8S rDNA). Bull calf liver had more DMCs associated with rDNA and the majority were hypermethylated in IUHT-B relative to IUCL-B. Expression of rDNA is regulated in part by CpG methylation; hypomethlyation of CpGs is associated with gene activation whereas transcriptionally silent genes are hypermethylated^83,84^. However, there can be hundreds of copies of rRNA genes across the genome, only a fraction of which are actively transcribed^85,86^. How methylation changes in a few copies of rRNA genes, as seen in the present study, impact rRNA transcription, ribosome biogenesis, and ultimately polypeptide formation is unclear. Thus, there are common patterns of DNA methylation changes induced by heat stress regardless of animal sex, age, or tissue type that may affect fundamental processes including cellular repair, oxidative defense, energy metabolism, and development.

DMGs in the present study consisted of a few to only 1 DMC, and those DMCs appeared to be associated with phenotype and function. Likewise, several studies have linked methylation changes at just one or two CpG sites with transcription activity and phenotype^87–90^. For instance, degenerate bovine embryos that failed to develop from the morula to the blastocyst stage had a higher level of methylation at a single CpG site upstream of the imprinted *PHLDA2* gene relative to embryos that reached the blastocyst stage, which was associated with upregulation of *PHLDA2*^88^. In another study, methylation of one specific CpG site within a 400 bp region of the oxytocin receptor gene (*Oxtr*) promoter inhibited transcription in an immortalized hypothalamic cell line^91^. A study on the role of epigenetics in developmental programming reported differences in methylation levels at specific CpG sites within the hippocampal glucocorticoid receptor (GR) gene between offspring reared by mothers with disparate maternal care behaviors^87^. Changes in methylation were associated with differences in histone H3-K9 acetylation and transcription factor binding to the *GR* promoter, with consequences for gene expression and offspring stress response^87^. These studies indicate that methylation patterns of one or a few critical CpG sites can drive changes in gene expression that can impact phenotype.

Although the general biological functions of differentially methylated genes and differentially expressed genes were similar in the present study, we found weak correlations between differential methylation and gene expression. Only two genes were both differentially methylated and differentially expressed, one of which is a novel bovine gene and another, *TCIRG1*, which is involved in adaptive immunity as a T cell regulator^92^. The precise regulatory role of DNA methylation in transcription is a current subject of debate. Methylation was originally considered to be transcriptionally repressive, particularly when occurring in promoter and enhancer regions, however the role of methylation in modulating transcription has proven to be substantially more complex^29,93^. For example, methylation of CpG rich promoters, which includes the majority of gene promoters, inhibits expression due to the binding of methylation-sensitive binding proteins and transcriptional repressors^94,95^. However, CpG poor promoters are often methylated irrespective of transcription activity^96–98^. It has also been noted that transcribed genes generally appear to be hypermethylated within the gene body^96,99,100^. The slight negative correlation between differential methylation of cytosines in the gene bodies and differential gene expression in our study seemingly contradicts these reports, but may be explained by the variability of gene expression relative to DNA methylation. According to common dogma, DNA methylation may be relatively stable throughout life, whereas patterns of gene expression vary drastically^31^. Thus, performing RNA sequencing at a particular time point provides a single snapshot of gene expression that may not necessarily correlate with DNA methylation patterns at that time. It is also possible that heat-induced changes in DNA methylation may not play a significant regulatory role in gene expression. Rather, heat stress may alter gene expression through other epigenetic modifications, such as histone modifications, chromatin remodeling, or microRNAs.

## Conclusions

Despite a few limitations of our study, including the 2-year interval between fetal exposure to heat stress and mammary tissue collection and the high culling rate of in utero heat stressed heifers which limited our pool of eligible biological replicates, we identified a substantial number of DMGs and DEGs between in utero heat stressed and cooled cattle. Those were involved in functions ranging from development, transcription regulation, and immunity to cell signaling, ATP synthesis, and oxidative defense. Dysregulation of these pathways may be associated with the observed morphological differences in the liver and mammary gland between in utero heat stressed and in utero cooled cattle and might contribute to the higher postnatal morbidity and reduced lactation performance of in utero heat stressed animals. A common pattern of DMGs between bull calf liver and heifer mammary gland was discovered despite obvious disparities in sex, tissue type, and age, suggesting that in utero heat stress may program different organs in a similar manner. Future studies evaluating the protein expression of DMGs and DEGs will provide further insight into the functional relevance of our findings.

## Materials and Methods

### Animals and experimental design

Trials were conducted at the University of Florida Dairy Unit (Hague, FL) in the summer months of 2014–2016 on a herd of Holstein cows. In 2014 and 2015, multiparous pregnant cows were dried off 46 days prior to expected calving date and randomly assigned to one of two groups; cooled (CL) and heat stressed (HT), based on mature equivalent milk production in the previous lactation. The CL and HT dams were housed on similar sized adjacent pens of the same shaded, sand-bedded, free-stall barn, but the cooled pen was equipped with fans and water soakers whereas the heat stressed pen lacked active cooling. Thus, the heat stressed group was exposed to the ambient environment with no heat abatement whereas the cooled group was exposed to the ambient environment but also provided heat abatement through fans and water soakers to facilitate evaporative cooling. In the cooled pen, fans ran continuously and water soakers turned on for 1.5 min in 6 min intervals when ambient temperature rose above 21.1 °C. The temperature humidity index (THI, Dikmen *et al*.^101^) of both sides of the barn was above 70 for the duration of the experiments in both 2014 and 2015^6^. Elevated respiration rate and rectal temperature are physiological indicators of heat stress in dairy cattle and other animals and were therefore used to assess treatment effectiveness^6,102,103^. Respiration rate (at 1400 h) and rectal temperature (at 1430 h) were recorded 3 times per week. Respiration rate was measured by counting the number of flank movements in one minute. Respiration rate and rectal temperature were significantly lower for CL relative to HT dams, confirming heat stress abatement in the cooled group and heat stress in the heat stressed group^6,102^.

Ten bull calves (n = 7 born in 2014, n = 3 born in 2015) and 7 heifers (all born in 2014) gestated by CL or HT dams were used for this study. These calves were exposed to the maternal treatment through the intrauterine environment during the last 6–7 weeks (~46 days) of fetal development. Thus calves belonged to one of four groups; in utero heat stressed heifers (IUHT-H, n = 3), in utero cooled heifers (IUCL-H, n = 4), in utero heat stressed bulls (IUHT-B, n = 5), and in utero cooled bulls (IUCL-B, n = 5). Bull calves were sacrificed by captive bolt stunning and exsanguination within 4 hours of birth, prior to first colostrum feeding. Heifers born in 2014 remained in the herd and were housed together as a group through their first lactation in summer 2016. The heifers all experienced the same environmental conditions and the same management regime, including separation from the dam at birth, routine vaccinations, and diet. Animal studies were approved by the Institutional Animal Care and Use Committee at the University of Florida (#201408505 and #201609371), and all experiments were conducted in accordance with their rules and regulations.

### Liver and mammary gland collection

Liver tissue was harvested from bull calves immediately after sacrifice, snap frozen in liquid nitrogen, and stored at −80 °C until methylation sequencing. Mammary gland biopsies were collected from heifers on day 21 of lactation, coinciding with the rising milk yield phase of the lactation cycle. Biopsies were performed using a stainless steel biopsy tool attached to a drill following the method described by^104^, with modifications by our group^18^. Mammary tissue was snap frozen in liquid nitrogen and stored at −80 °C until methylation sequencing. An additional 5 mm piece of liver and mammary tissue was placed in tissue cassettes overnight at 4 °C in 4% paraformaldehyde, subsequently rinsed in a graded series of ethanol, and paraffin embedded. Tissues were sectioned at 5 μm onto slides coated with poly-L-lysine and stained with hematoxylin and eosin to visualize morphology.

### Quantification of histological sections

Three images per tissue section were photographed with an EVOS XL Core imaging system (Advanced Microscopy Group, Bothell, WA) using a 20X or 40X objective (total tissue area was 573,573.619 μm^2^ and 95,908.32 μm^2^, respectively). The number of alveoli in mammary tissue (at 20X) and the number of cells in liver tissue (at 40X) were quantified for each tissue image using the Point Picker plugin (Biomedical Imaging Group, Swiss Federal Institute of Technology Lausanne, Lausanne, Switzerland) for Image J software (National Institutes of Health, Bethesda, MD). Alveoli were discriminated from other structures by their rounded hollow lumen enclosed by a single layer of mammary epithelial cells. Alveoli area (at 20X) was quantified using the free-hand drawing tool in Image J and expressed as μm^2^. Mammary ducts were not included in alveoli counts and area measurements. Differences between IUHT and IUCL groups were analyzed by two-tailed unpaired T-tests using SAS v. 9.4 (SAS Institute, Cary, NC). Heifers and bulls were analyzed separately. P-values < 0.05 were considered statistically significant. Data are presented as the mean ± standard error of the mean (SEM).

### DNA isolation and methylation sequencing

DNA was isolated from 50 mg of liver and mammary tissue with the FitAmp^TM^ General Tissue Section DNA Isolation Kit (Epigentek, P-1003). DNA methylation was analyzed by double restriction enzyme reduced representation bisulfite sequencing (RRBS). RRBS provides methylation resolution at the single nucleotide level from a subset of the genome^105^. Despite low genome coverage, on average 2%^106,107^, RRBS enriches for CpG rich areas, including the majority of CpG islands and gene promoters and to a lesser extent introns, exons, CpG shores, and CpG shelves^106^. Thus, RRBS is a powerful method that is broadly used for the assessment of DNA methylation patterns.

First, DNA was digested with MspI restriction enzyme for 2 hrs at 37 °C followed by digestion with Taq^α^I restriction endonuclease at 65 °C. DNA fragments < 300 bp were selected and used for bisulfite treatment with the Methylamp DNA Conversion Kit (Epigentek, P-1001). Conversion efficiency was determined by rtPCR using two primer pairs; the first for β-actin against bisulfite converted DNA and the second for GAPDH against unconverted DNA. The same bisulfite converted DNA sample was run with both primer pairs. DNA was >98% converted. Standard lllumina sequencing adaptors were ligated onto the bisulfite treated DNA fragments using a random probing method. Library amplification was achieved using indexed primers and analyzed on a Bioanalyzer and with a KAPA Library Quantification Kit according to the manufacturer’s protocol (KapaBiosystems, MA, USA). Purified DNA (15 nM of sample) libraries were sequenced using the Illumina HiSeq. 4000 system, generating 50 bp single-end reads. Reads from one bull calf (IUHT-B) could not be aligned to the reference genome and was therefore excluded from further analysis. RRBS data can be accessed by NCBI GEO with accession number GSE119445.

### Bioinformatics analysis

Quality control of the Illumina raw reads was performed using FastQC software (version 0.11.2, Babraham Bioinformatics, UK). Low quality base removal and adapter trimming was carried out using Trim Galore (version 0.2.7, Babraham Bioinformatics, UK) in the RRBS-specific mode. A Sanger Phred score of 20 was used as the cut-off criterion for removal of low quality reads. The 3′ Illumina adapter was trimmed and any reads shorter than 20 bp were removed. Trimmed reads were mapped to the bovine genome assembly UMD3.1.1 using Bismark software (version 0.16.1, Babraham Bioinformatics, UK), which utilizes the Bowtie short read aligner^108^. The options –n 1 and pbat were selected, which permits up to one mismatch in the seed region and indicates the sequencing library was constructed following a post-bisulfite (PBAT) protocol, respectively. Methylation information was extracted using the Bismark methylation extractor (version 0.16.1, Babraham Bioinformatics, UK) at the base resolution. The ignore 6 option was selected to ignore the first 6 bp of each read to reduce methylation bias typically observed in PBAT libraries. A minimum read coverage of 5 and minimum quality score of 20 at each base position was applied. Sequencing read counts and levels of methylation were calculated with the methylKit package in R. Only cytosines with more than 10 reads were analyzed further. Differentially methylated cytosines (**DMCs**) between IUHT-H and IUCL-H and between IUHT-B and IUCL-B were identified via logistic regression with a cut-off value of 15% methylation difference and a *q*-value < 0.2. DMCs that had a lower level of methylation in the IUHT groups relative to the IUCL groups were considered to be hypomethylated. Conversely, DMCs that had a higher level methylation in the IUHT groups relative to the IUCL groups were considered to be hypermethylated. Principal component analysis was used to distinguish groups based on intra- and inter-group variability in methylation levels. Differentially methylated genes (**DMGs**) have at least 1 differentially methylated cytosine. Pathways, biological processes, and molecular functions of DMGs were identified using Kyoto Encyclopedia for Genes and Genomes (KEGG) database, Medical Subject Headings (MeSH) database, and Gene Ontology database (Gene Ontology Consortium).

### RNA isolation and RNA-sequencing

In order to explore regulation of gene expression by DNA methylation we conducted correlations between differential methylation at the cytosine level and differential gene expression using the mammary gland tissue. A portion of the mammary biopsies from all IUHT-H and IUCL-H were stored at −80 °C in RNALater until RNA extraction for RNA sequencing. Briefly, RNA was extracted from mammary tissue using the RNeasy Plus Universal Mini Kit (Qiagen, Valencia, CA) and RNA concentration was determined using a NanoDrop instrument (ND-2000, ThermoFisher Scientific, Waltham, MA). RNA quality was assessed using the Agilent 2100 Bioanalyzer (Agilent Technologies, Inc., Santa Clara, CA). RNA with 28S/18S > 1 and an RNA integrity number ≥7 were used for library construction. RNA-Seq library was constructed using NEBNext® Ultra™ RNA Library Prep Kit for Illumina (New England Biolabs, Ipswich, MA) following manufacturer’s recommendations. Libraries were sequenced on the Illumina HiSeq. 3000 platform generating 100 paired-end reads.

Quality control of sequencing reads was conducted using FastQC software. Tophat (v2.0.13) software was used to map reads to the bovine reference genome (bosTau7). Identification of differentially expressed genes (DEGs) was accomplished using the R package edgeR (v.3.4.2), using a *P*-value cut-off of 0.01. Gene set enrichment analysis for differentially expressed genes was performed in R using the Gene Ontology database (Gene Ontology Consortium). Relationships between differentially expressed genes and differentially methylated cytosines associated with those genes were analyzed through Pearson correlation in R software. Separate correlations were run for the overall DMCs and a subset of the DMCs based on genomic location (upstream, downstream, and gene body) to determine if DMCs associated with particular genomic regions have different influences on gene expression. RNA-seq data can be accessed by NCBI GEO with the accession number GSE119216.

## Electronic supplementary material

Supplementary Information

Table 1

Table 2

Table 3

Table 4

Table 5
