# Supplementary material for: In Utero Heat Stress Alters the Offspring Epigenome
Source: Sci Rep. 2018 Oct 2;8:14609. doi: 10.1038/s41598-018-32975-1 (PMC6168509; doi:10.1038/s41598-018-32975-1)
Supplement: Supplementary file 1 — Supplementary Information [file 41598_2018_32975_MOESM1_ESM.docx]

***Supplementary Information***

**In Utero Heat Stress Alters the Offspring Epigenome**

**A. L. Skibiel, F. Peñagaricano, R. Amorín, B. M. Ahmed, G. E. Dahl^*^, J. Laporta^*^**

***Correspondence:** J. Laporta: [jlaporta@ufl.edu](mailto:jlaporta@ufl.edu), G. E. Dahl: [gdahl@ufl.edu](mailto:gdahl@ufl.edu)


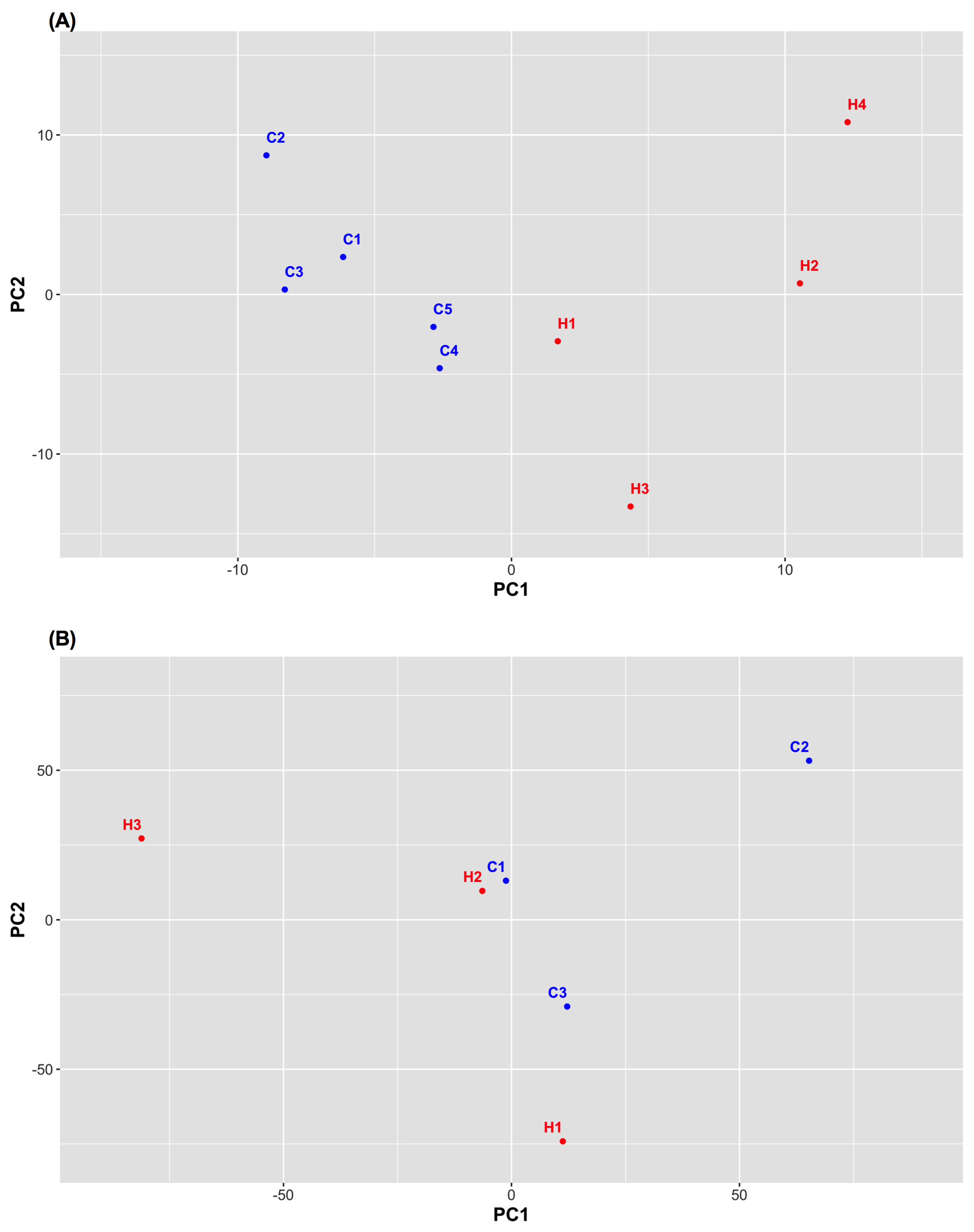


**Supplementary Figure 1.** Principle components analysis of differentially methylated cytosines between in utero heat stressed and in utero cooled cattle. (A) Bull calf liver; (B) Heifer mammary gland. Bull calves and heifers were born to dams that were heat stressed (access to shade only) or cooled (access to shade, fans, and water soakers) during late gestation (approximately 46 days). Data points correspond to individuals; C denotes in utero cooled animals whereas H denotes in utero heat stressed animals.

**Supplementary Table 1.** Chromosomal location and methylation information for differentially methylated cytosines in bull calf liver and heifer mammary DNA.

**Supplementary Table 2.** Genomic location and methylation information for differentially methylated cytosines in bull calf liver and heifer mammary DNA.

**Supplementary Table 3.** Differentially methylated genes common to bull calf liver and heifer mammary gland.

**Supplementary Table 4.** Differentially expressed genes in the mammary gland of in utero heat stressed (IUHT-H) versus in utero cooled heifers (IUCL-H)

**Supplementary Table 5.** Pathways, biological functions, and molecular processes of differentially expressed genes in the mammary gland of in utero heat stressed (IUHT-H) versus in utero cooled heifers (IUCL-H).
